# Oblique effects with multisegment spectacle lenses: 2. Ray tracing to determine power corrections

**DOI:** 10.1111/opo.13477

**Published:** 2025-03-10

**Authors:** David A. Atchison, W. Neil Charman, Matt Jaskulski

**Affiliations:** ^1^ Centre for Vision and Eye Research Queensland University of Technology Brisbane Queensland Australia; ^2^ Faculty of Biology, Medicine and Health, School of Health Sciences University of Manchester Manchester UK; ^3^ School of Optometry Indiana University Indiana USA

**Keywords:** multisegment lenses, myopia, myopia control, peripheral refraction

## Abstract

**Purpose:**

Part 1 of the study investigated image quality associated with oblique incidence of light on a multisegment lens (Hoya MiyoSmart) intended to treat myopia development. Part 2 investigates power corrections associated with oblique incidence.

**Methods:**

Modelling and ray tracing were carried out with lenses of −4 D distance power and, to a lesser extent, +0.25 D. Ray tracing simulations were done for the lens by itself, an eye model by itself and the combination. These simulations were for the static situation of peripheral vision when the eye looks through the lens centre and for central (foveal) vision when the eye rotates to look at objects away from the lens optical axis. The outcome was power correction of the optics, that is, the difference between the nominal power of the distance correction provided by the carrier lens under specific conditions and the actual power. This was determined across the field to about 45° and across the pupil for certain field angles. Most investigations were performed for distance vision, but some were for near objects with an accommodating version of the model eye.

**Results:**

Ignoring intended multisegment effects, the quality of optics associated with peripheral vision was poor. There was considerable astigmatism (cylinder) across the field and high variation in astigmatism across the pupil. The added effects of the lens and eye were similar to those of their combination. For the accommodated eye model with an object at 250 mm, results were similar to those obtained with the unaccommodated model viewing a distant object. For foveal vision with the rotating eye, optics were relatively good with lower levels of astigmatism than for peripheral vision.

**Conclusion:**

The results of Part 1, finding considerable effects of the obliquity of incidence associated with peripheral vision and with foveal vision for the rotating eye, were supported by the power corrections.

**Key points**

- Optical quality of multisegment lenses, used for the management of myopia, is affected considerably by oblique incidence at the lens and eye.
- Oblique incidence occurs in peripheral vision, with the eye looking through the centre of the lens, and in foveal vision of the rotating eye.
- Peripheral vision produces high levels of astigmatism, through both the distance and segment parts of the lenses, while the rotating eye causes much smaller effects.

## INTRODUCTION

Multisegment (MS) spectacle lenses^1^ are being used widely in attempts to control the age‐dependent increases in axial length and myopia that have become common in many parts of the world. Several designs are now available and have been reported to reduce these unwanted increases. All involve modification of the image falling on the peripheral retina. Further development of MS lenses, however, requires a better understanding of the optical effects of the current designs on the retinal image. In a recent paper, it was shown that the obliquity of the ray bundles responsible for peripheral vision during MS lens wear may be important, since the associated increases in aberrations considerably degrade the quality of images of point objects.^2^

The purpose of the present paper is to progress the work of Charman et al.^2^ by determining the power corrections of the Hoya MiyoSmart (hoyavision.com) multisegment lenses away from the optical axis. Ray tracing will be done under two situations—in peripheral (stationary) vision when the eye looks through the centre of the lens, thus avoiding the lenslets as intended, and in central vision when the eye rotates to look directly at an object through the lens periphery. Some results have been presented previously.^1^

## METHOD

### Lenses

As considerable details of the Hoya MiyoSmart lenses have been provided by Charman et al.,^2^ only a brief description is given here. The lenses are made of polycarbonate (refractive index 1.586) and have a base curve of 3.0 D (1.523 index). A back vertex power of −4.00 D was used in most of the present study, although some investigation was done with a +0.25 D power. The circular lenslets, each with a nominal diameter of 1 mm and a power of +4 D, are arranged on the anterior surface of the carrier lens into nine hexagons around a central distance zone. For the present work, it was assumed that the lenses were positioned so that two opposite apices of the hexagons were always aligned vertically on the lens. Lenses were modelled as ‘non‐sequential’ components in Zemax OpticStudio version R 2.01 (Ansys Zemax, ansys.com/products/optics/ansys‐zemax‐opticsstudio).

### Modelling

As in the earlier paper,^2^ modelling was performed for the lens both without and with the model eye. In the lens‐alone case, a stop was placed at the location of the entrance pupil of the model eye, 11.86 mm in front of the eye's centre of rotation and 15.14 mm behind the lens. The stop diameter was assumed to be 5.0 mm, corresponding to the typically large pupils of children. The image surface was at the paraxial focus for a distant object. For peripheral (static) vision, the image surface's centre of curvature was the stop of the lens. For the rotating eye situation, the image surface's centre of curvature was the centre‐of‐rotation of the eye; this surface is usually called the far point sphere. For both the peripheral and the rotating situations, ray tracing proceeded through the lens to the centre‐of‐rotation, 27 mm behind the lens back surface. The centre‐of‐rotation was a ‘co‐ordinate break’, about which the rest of the optical system could be rotated as appropriate. Ray tracing proceeded backwards 11.86 mm to the stop and then forwards to the second principal plane and the image surface.

For ray tracing of the eye by itself or of the combined lens and eye, the Atchison model eye was used.^3^, ^4^ This has some parameters dependent linearly on the spectacle refraction, while others are dependent upon accommodation, and vitreous depth is affected linearly by both. Ray tracing proceeded through the lens to the centre‐of‐rotation 27 mm from the lens, then backwards 15 mm to the cornea, through the cornea to the stop (3.7 mm inside the eye for the unaccommodated eye), through the eye lens, to the second principal plane of the system and to the retina.

### Determining power corrections

Field angles were chosen to analyse either horizontal or vertical meridians. For ray tracing involving the rotating eye, the image field position is zero. Nevertheless, an object field angle can be specified as this is relevant to the lens in front of the eye.

The main interest in this study was powers and reduced vergences when rays or ray beams were incident obliquely at the lens surfaces. Results were expressed in terms of field power maps and pupil power maps after refraction at the second principal plane.

**FIGURE 1:**
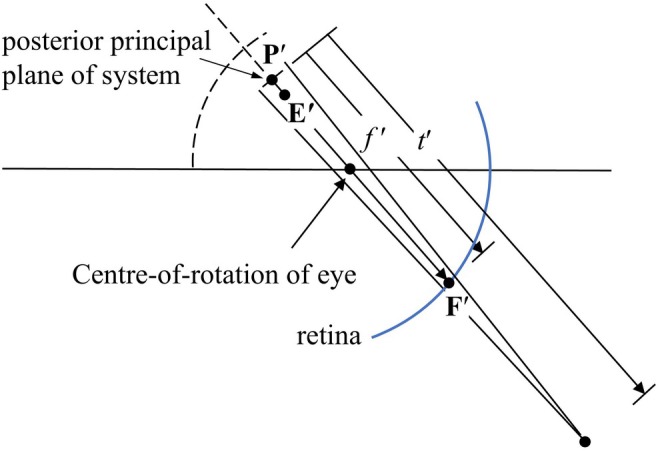
Determination in Zemax of tangential power in rotatory vision for a lens–eye combination. P′ is back principal point, F′ is ideal back focal point at retina, E′ is exit pupil centre, P′F′ is ideal back focal length *f′* along the chief ray between the back principal plane and the retina and *t′* is the tangential focal length.

To present the field power maps as power corrections, the chief ray must be traced from its intersection with the last refracting surface to the second principal plane of either the lens alone or of the lens–eye combination. The sagittal and tangential corrections *C*_s_ and *C*_t_ are

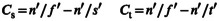

 where *f*′ is the distance from the second principal plane to the image surface along the chief ray, *s*′ and *t*′ are the sagittal and tangential focal lengths and *n*′ is the refractive index of the final media with values of 1.0 and 1.336 for the lens and lens–eye combination, respectively (Figure 1). In practice, *f*′ was determined for several field angles and a second‐order fit was calculated.

In addition to the sagittal and tangential corrections, on occasions, a sphero‐cylinder correction is given where the spherical portion in dioptres sphere (DS) is the same as the sagittal correction and the cylinder correction in dioptres cylinder (DC) is the tangential correction minus the sagittal correction.

To present the pupil power maps as power corrections, a similar process was followed as for the field power maps, but here a range of *f′* values was determined across the pupil for a field position of interest, with each pupil position having its own ‘chief’ ray.

Some results will be given for the case of near, rather than distant, objects. Because the determination of powers in Zemax requires that the object is at infinity, the above method for determining power corrections had to be modified in the case of an accommodated schematic eye with a near object. A paraxial lens (a lens without aberration) was placed immediately in front of the MS lens. It had approximately a 250 mm focal length to match an object the same distance in front of the lens. The second principal plane is affected by the introduction of the additional lens, and care was taken to re‐reference the determined focal lengths to the second principal plane without the additional lens.

In spectacle lens design where the lens is considered by itself, corrections are determined relative to the vertex sphere, which touches the back vertex of the lens and rotates about the eye's centre‐of‐rotation. In a few cases, corrections that would be placed immediately in front of the spectacle lenses, similar to the way a refraction is done clinically, were compared with those corrections referenced to the second principal plane of the optical system. The former were found by placing paraxial X–Y lenses (ideal thin toroidal lenses) at the plane passing through the vertex of the front surface of the spectacle lens. The X–Y lenses were decentred to be along chief rays and orientated at right angles to them. The ‘Optimise’ routine in Zemax was used to minimise the sizes of point spread functions by changing powers of the X–Y lenses and thus determining the corrections.

## RESULTS

### Peripheral optics, lenses alone

**FIGURE 2:**
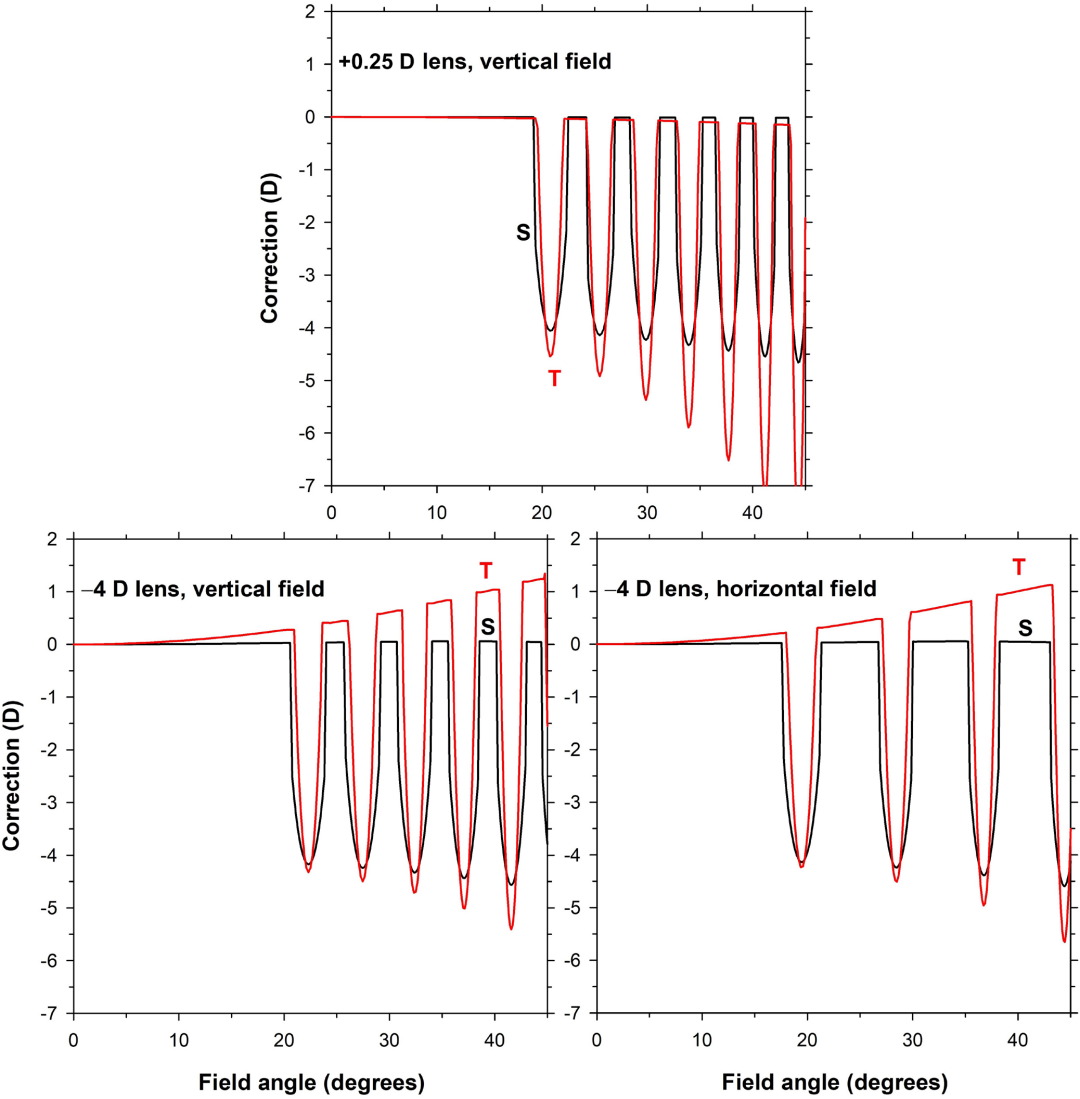
Corrections for +0.25 D and −4 D multisegment lenses in peripheral vision. (Top) +0.25 D lens, vertical field. (Bottom) −4 D lens for vertical field (left) and horizontal field (right). Black lines and red lines are for sagittal (S) and tangential (T) corrections, respectively. For this figure and for Figures 4, 5 and 9, discontinuities in corrections at carrier/lenslet boundaries have been smoothed.

Figure 2 (top) shows the corrections of a +0.25 D lens along the vertical field, referenced to the second principal plane of the lens. As expected for such a low‐powered lens, the corrections through the lenslet‐free, central part of the lens out to a field angle of about 19° are minimal. The correction through the first lenslet hexagon (~21°) is greater than needed to correct the lenslet power, at −4.0 DS/−0.5 DC × 180. The tangential correction is more negative than the sagittal correction. The correction increases quickly into the field, and for a ray bundle passing through the lens at the sixth hexagon (corresponding to a field angle of ~41°), it is −4.5 DS/−2.7 DC × 180.

Figure 2 (bottom) shows the corrections of a −4 D lens along the vertical (left) and horizontal (right) fields. The corrections are referenced to the second principal plane of the lens; referencing to the vertex sphere of the lens makes <0.1 D changes in most circumstances. The inclusion of the horizontal field (Figure 2, bottom right) is to show that the repetition of the lenslets occurs nearly twice as frequently vertically than horizontally because of the way that the hexagons have been arranged. The distance part of the lens shows peripheral power correction of 0.0 DS/+1.3 DC × 180 at a 45° field angle. As in the case of the +0.25 D lens (Figure 2, top), the correction through the hexagons increases quickly with an increase in visual field, although not as quickly as for the +0.25 D lens. If account is taken of the reduced rate at which the hexagons appear into the field for the negative lens, then the change appears closer, for example, comparing the fifth hexagon, the corrections are −4.4 DS/−2.1 DC × 180 for the +0.25 D lens (~38°) and −4.6 DS/−0.5 DC × 180 for the −4 D lens (~42°). These corrections are quite different in terms of the cylinder, but it should also be considered that −0.5 DC for the −4 D lens is a −1.8 DC change from that occurring at the distance portion.

### Peripheral optics, eyes alone

**FIGURE 3:**
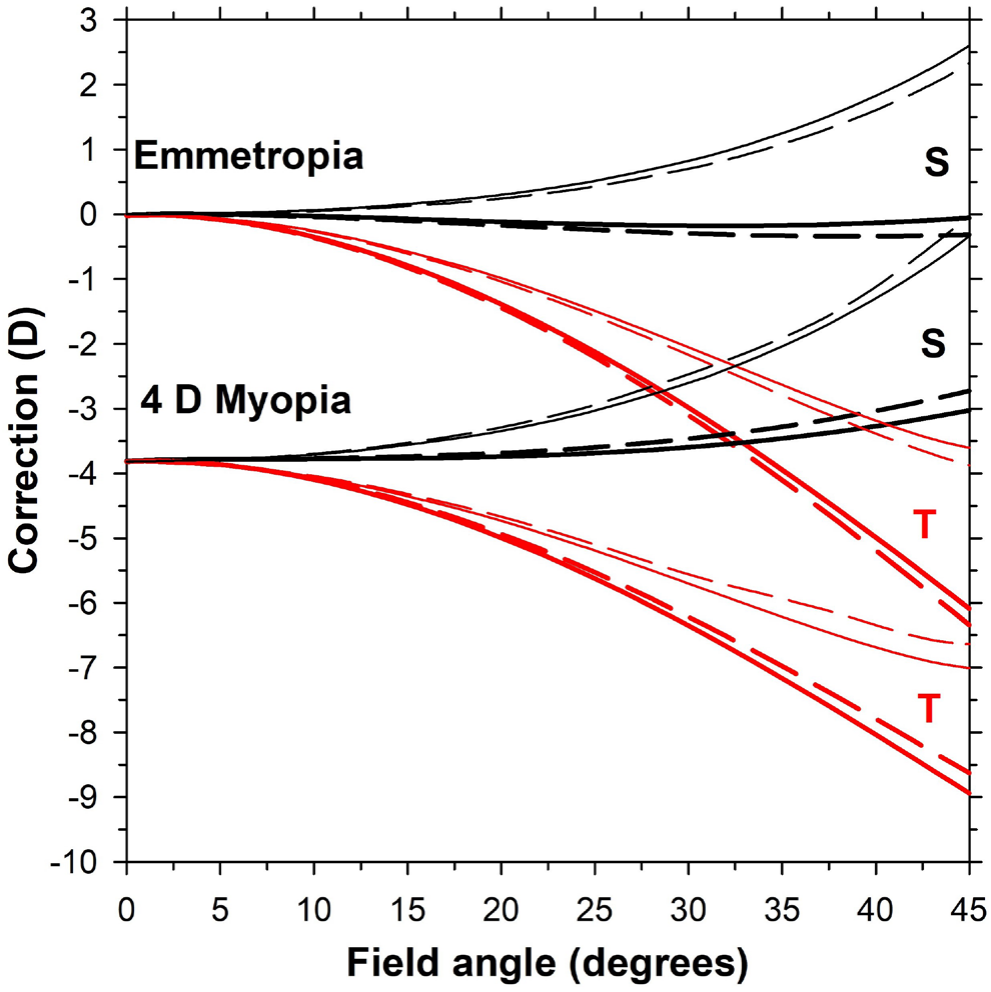
Corrections for emmetropic and 4 D myopic schematic eyes in peripheral vision. Thick lines and thin lines indicate corrections referenced to the second principal plane of the lens/eye combination and to the front of the eye, respectively. Black lines and red lines are for sagittal (S) and tangential (T) corrections, respectively. Solid lines and dashed lines are for vertical and horizontal visual fields, respectively.

Figure 3 shows corrections of emmetropic and 4 D myopic model eyes without lenses in front of them. These are shown both for corrections referenced to the second principal plane of the eye and to the front of the eye. When results are referenced to the second principal plane (thick lines), the emmetropic eye has flat sagittal plots and tangential plots with negative corrections amounting to about −4 D at a 45° field angle. Differences between vertical and horizontal fields do not exceed 0.5 D. Referencing corrections to the front of the eye (thin lines) moves the S and T corrections in the positive direction by approximately 2.5 D at a 45° field angle. Corrections for the 4 D myopic eye are shifted in the positive direction relative to those for the emmetropic eye, the shift amounting to about 2 D at a 45° angle.

Comparing these results with the distance part of the −4 D lens alone (Figure 2, bottom), the following points should be noted:

(i) The sagittal corrections are more positive than the tangential corrections, which is opposite to that occurring for the lens alone.
(ii) The cylinders are much higher than that occurring for the lens alone, for example, at 45° field, the 4 D myopic eye has ~−6.0 DC while the lens has +1.3 DC.

The method of determining peripheral correction affects the eye alone, but is of negligible influence for the lens alone.

### Peripheral optics, eye and lens combined

**FIGURE 4:**
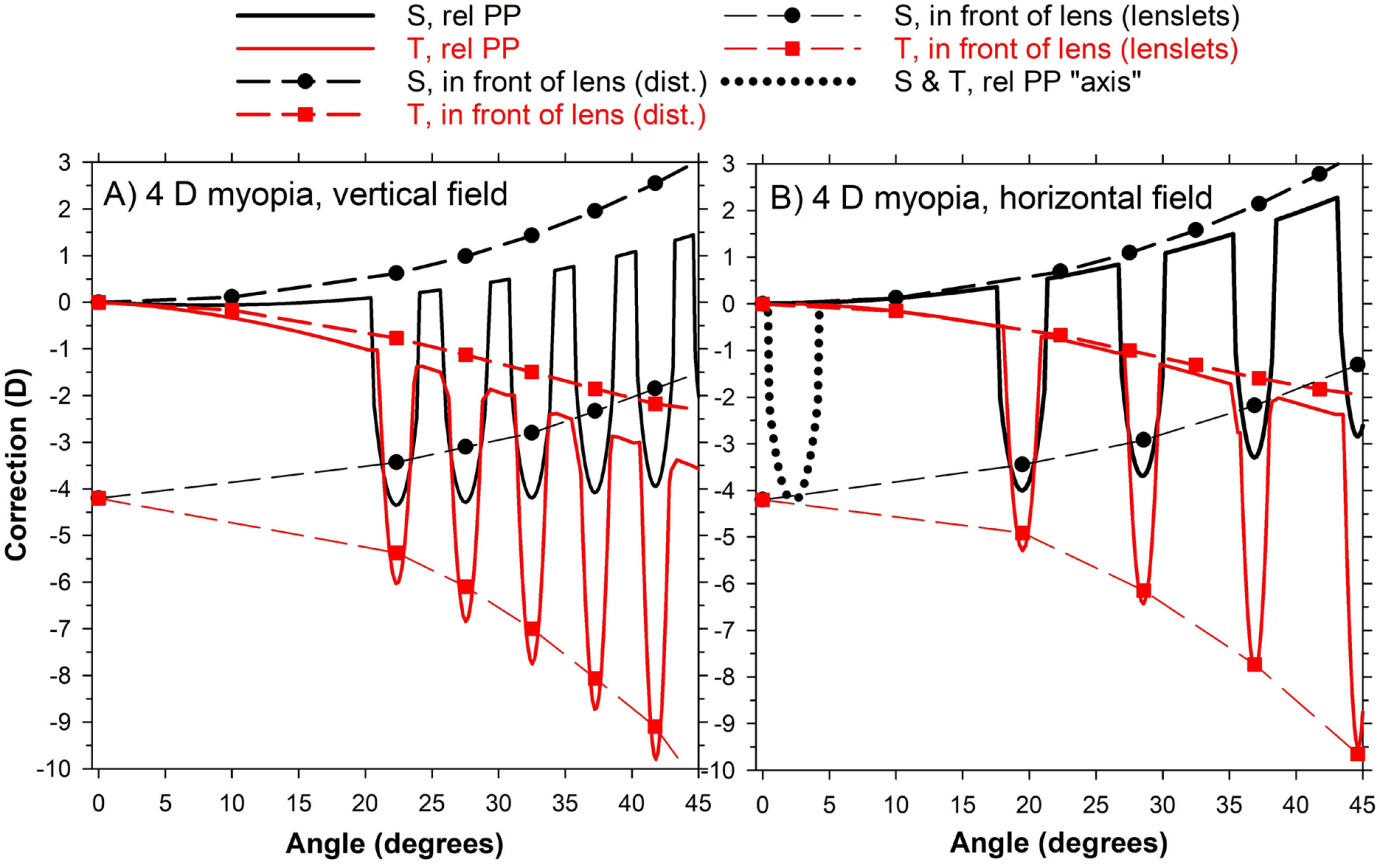
Corrections in peripheral vision for a 4 D myopic schematic eye, combined with a −4 D multisegment lens, for (A) vertical visual field and (B) horizontal visual field. Solid lines and dashed lines indicate corrections referenced to the second principal plane (PP) of the eye and to the front of the lens, respectively. Black lines and symbols are for sagittal (S) corrections, and red lines and symbols are for tangential (T) corrections. The corrections referenced to the front of the lens have been determined only at discrete positions. The dotted black line indicates the correction if a lenslet was placed close to the lens optical axis. rel, relative

**FIGURE 5:**
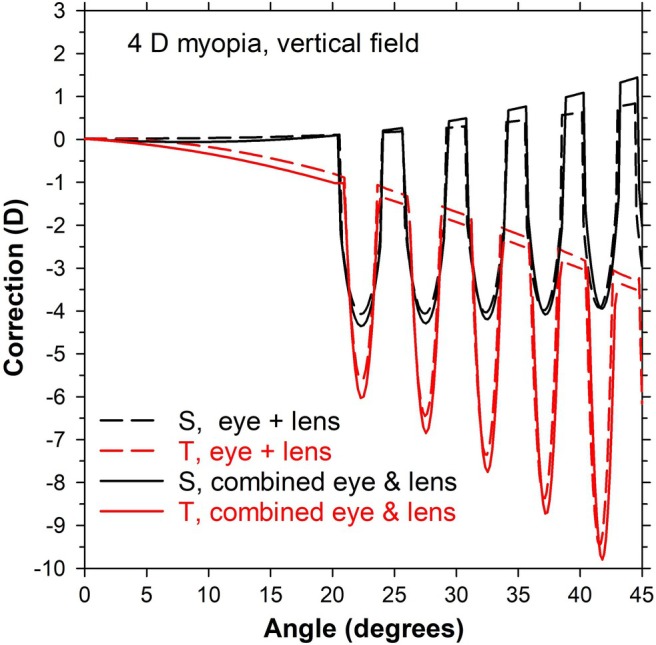
Corrections in peripheral vision along the vertical field for 4 D myopic schematic eye, combined with a −4 D multisegment lens. The sagittal (S) and tangential (T) ‘eye + lens’ plots are obtained by adding the corrections of the lenses in Figure 3 bottom to the lens corrections referenced to the second principal plane (PP) (after subtracting the on‐axis correction). The S and T ‘combined eye & lens’ plots are taken from Figure 4 (S, rel PP; T, rel PP).

Here, it is assumed that the optical axes of the carrier lens and the eye coincide. Figure 4 shows corrections for the −4 D lens combined with the 4 D myopic eye along both vertical and horizontal fields. Comparing Figure 4 with Figures 2 and 3 shows that the optics of the combined eye and lens are approximately equal to the added separate effects of the lens and the eye; this is confirmed in Figure 5. This combination produces very high corrections, for example, at 32.5°, corresponding to the chief ray passing through the third hexagon, the correction is −4.2 DS/−3.6 DC × 180 (mean sphere −6.0 D). The correction of the distance (carrier) region would be +0.6 DS/−2.8 DC × 180, so the additional effect due to the lenslet is −4.8 DS/−0.8 DC × 180, compared with the specified add of +4.1 D, indicating that the additional cylinder produced through the lenslets is considerably less than that produced through the distance (carrier) part of the lens. Other things to note are:

(i) Results referenced to the front of the lens are more positive (or less negative) than those referenced to the principal plane, although the differences are less than for the eye alone.
(ii) The angular extent of the lenslets decreases with an increase in visual field angle. See, in particular, Figure 4B for the change in dimension from the artificial lenslet near the axis and the third lenslet outwards.

**FIGURE 6:**
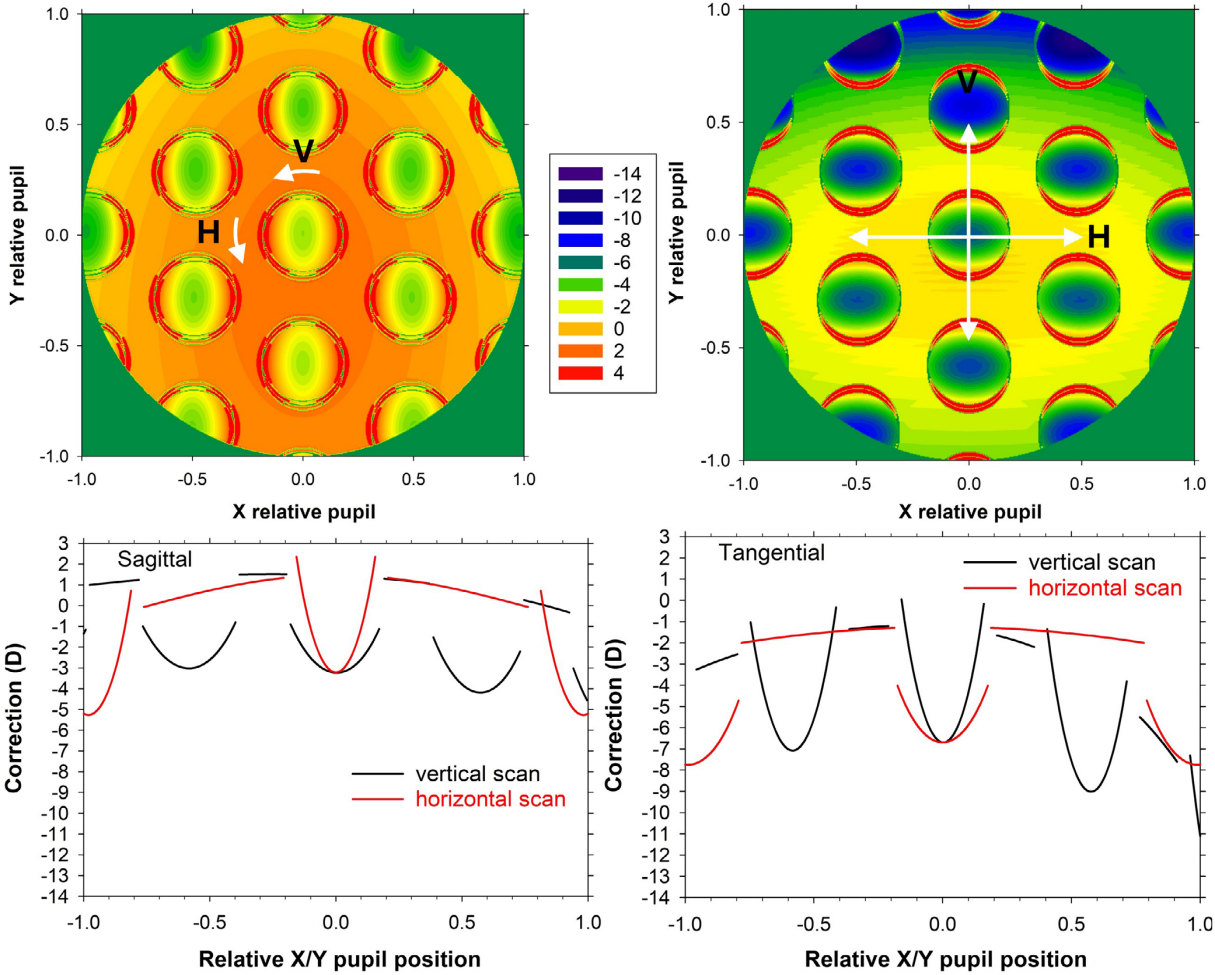
Pupil powers for the 4 D myopic schematic eye with a −4 D multisegment lens, corresponding to a 32.5° vertical field. Top: Sagittal (left) and tangential (right) two‐dimensional maps; Bottom: Sagittal (left) and tangential (right) scans along the vertical and horizontal field meridians.

Figure 6 shows sagittal and tangential power corrections across the diameter of a 5‐mm stop when the third hexagon is imaged through the middle of the pupil (visual field angle 32.5°). The distance coordinate is the relative pupil position, that is, the actual distance from the centre of the pupil, divided by the pupil radius (2.5 mm). In the way that the ray tracing is set up (Figure 1 left of Charman et al.^2^), the object is below the eye, and the rays passing near the top of the pupil are closer to the axis than rays passing near the bottom of the pupil, with the former at greater obliquities to the lens axis. Note that there is symmetry about the vertical pupil meridian. The power variation is considerable, more so for tangential than sagittal correction and greater along the vertical than the horizontal meridian. For example, the correction at the centre of the pupil is −3.2 DS/−3.4 DC × 180, while at the second hexagon (1.5 mm above centre) and the fourth hexagon (1.5 mm below centre), it is −4.2 DS/−4.8 DC × 180 and −3.0 DS/−4.1 DC × 180, respectively.

### Optics of foveal vision with the rotating eye, eye and lens combined

**FIGURE 7:**
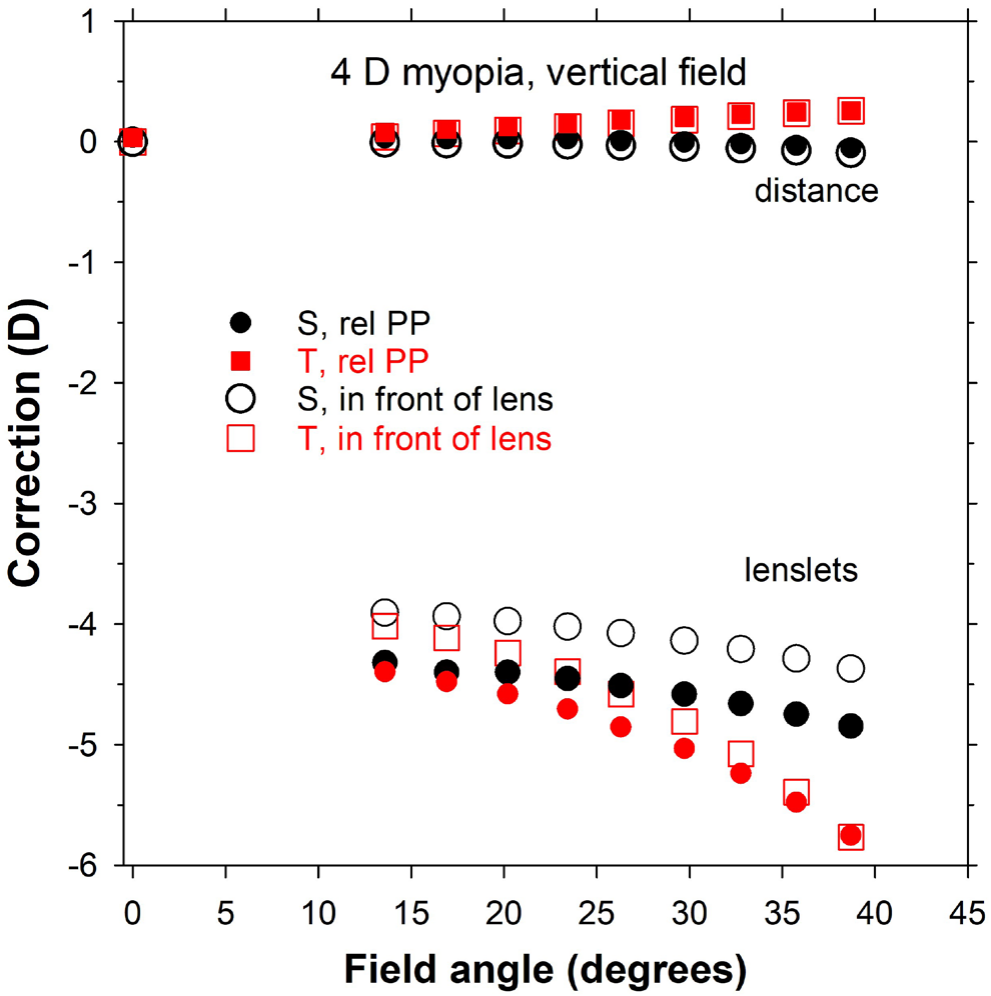
Corrections for the rotating eye for 4 D myopic schematic eyes combined with a −4 D multisegment lens. Filled and open symbols indicate corrections referenced to the second principal plane (PP) of the eye and to the front of the spectacle lens, respectively. Red and black symbols are for sagittal (S) and tangential (T) corrections, respectively. The corrections have been determined only at discrete positions. rel, relative.

For foveal vision, corresponding to the eye rotating behind the lens, there is no need to evaluate the eye by itself as the optics will not change with rotation angle. Here, the eye rotates vertically about a horizontal axis from an initial position, in which its optical axis corresponds to that of the carrier lens, to view through the corner lenslets of successive lenslet hexagons. Figure 7 shows foveal corrections for the 4 D myopic eye rotating behind the −4 D lens. Only discrete points are shown. Results are shown both for corrections referenced to the second principal plane (filled symbols) and to the front of the multisegment lens (open symbols). The corrections are negligible for the distance (carrier) part of the lens, for example, +0.2 DS/−0.3 DC × 90 (mean sphere +0.1 D) at a 35.6° field angle (rotation 31.7°). For the lenslets, the results referenced to the front of the lens are more positive (or less negative) than those referenced to the principal plane. For the latter, the correction through the eighth hexagon at 35.6° is −4.8 DS/−0.7 DC × 180 (−5.1 D mean sphere). The cylinder is much smaller than that occurring at similar angles in peripheral vision, for example, −4.2 DS/−3.6 DC × 180 at 32.5° (Figure 4, left).

**FIGURE 8:**
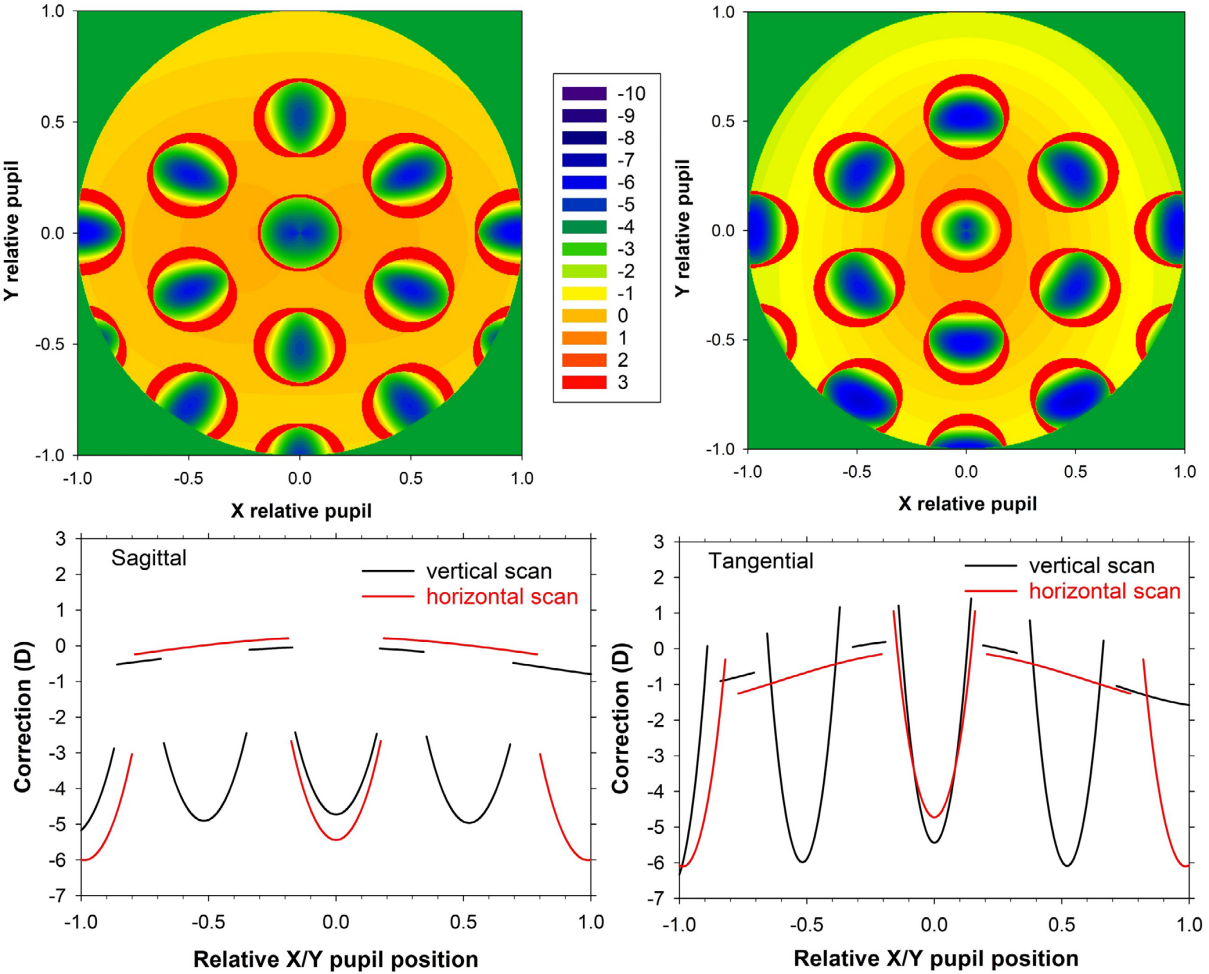
Pupil powers for the 4 D myopic schematic eye with a −4 D multisegment lens, corresponding to a 35.6 vertical field angle (31.7° eye rotation). Top: Sagittal (left) and tangential (right) two‐dimensional maps; Bottom: Sagittal (left) and tangential (right) scans along the vertical and horizontal directions.

Figure 8 shows sagittal and tangential corrections across a 5‐mm stop when the eighth hexagon is imaged through the middle of the pupil (visual field angle 35.6°, 31.7° rotation). In the way that the ray tracing is set up (Figure 1 right of Charman et al.^2^), the object is above the eye and the rays passing near the top of the pupil are further from the optical axis than rays passing near the bottom of the pupil, with the former at smaller obliquities to the lens optical axis. There is symmetry about the vertical pupil meridian. The power variation is less than occurs for peripheral vision at a similar angle (Figure 5), particularly if the distance and lenslet regions are considered separately. The variation across the distance regions is, in part, a manifestation of spherical aberration. Variation is greater for tangential than sagittal correction and is similar along the vertical and horizontal meridians.

### Peripheral optics, eye and lens combined, for the accommodating eye and near vision

**FIGURE 9:**
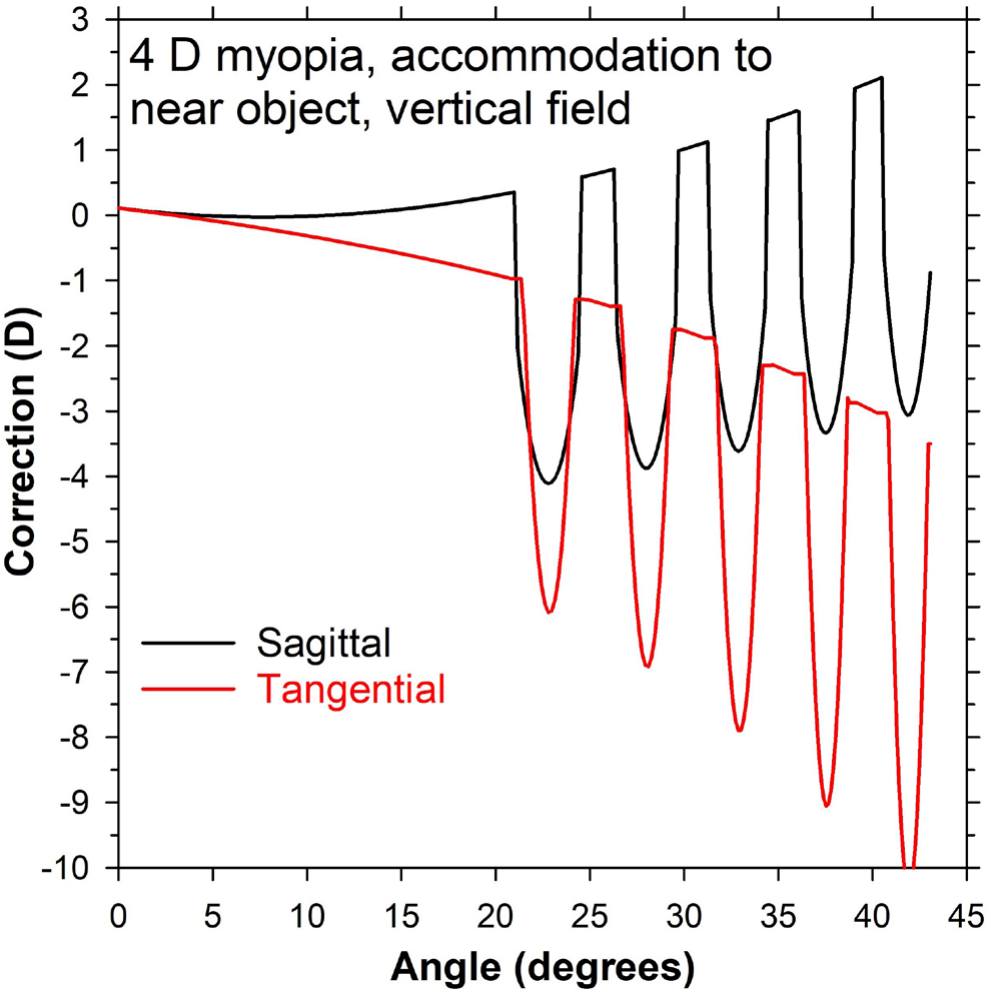
Correction in the vertical visual field for the 4 D myopic schematic eye with 4 D accommodation and a near object 221 mm in front of the −4 D multisegment lens. Results are referenced to the second principal plane of the lens/eye combination. Black and red lines are for sagittal and tangential corrections, respectively.

This is a repeat of the analysis for peripheral vision, but for an object 221 mm in front of the lens, corresponding to 4.53 D spectacle accommodation and 3.93 D ocular accommodation. The results in Figure 9 are similar to those occurring for the distance case (Figure 4, left). This is not surprising as the accommodating eye models were designed to have similar peripheral corrections as the unaccommodated eyes.^4^ There are small differences, most noticeably with the sagittal corrections being shifted in the positive direction.

## DISCUSSION

This theoretical study investigated the power corrections associated with the Hoya MiyoSmart multisegment lens used for the treatment of myopia. This was done for peripheral (stationary) vision, when the eye is looking through the centre of the lens, and for foveal central vision when the eye is rotated to look at objects through the lens periphery. As appropriate, results were presented for the lens alone, the eye alone, and the combination of the eye and the lens. An adult eye model was used for which several parameters vary according to the level of myopia and accommodation. Most results were presented for a 4 D, unaccommodated myopic eye.

While the parameter variations are limited, for example, fixed stop and centre‐of‐rotation positions, use of an adult model rather than a children's model, most results for a 4 D myopic eye and ignoring decentrations, tilts and asymmetry of lens and eye surfaces (except for a biconic retina), there is enough information to make some broad conclusions. In general, obliquity of ray bundles through either the lens alone or the complete lens–eye system is associated with the introduction of power corrections which may have a magnitude large enough to significantly affect vision in the peripheral field.

The quality of the optics associated with peripheral vision (when the eye maintains fixation along the carrier lens axis through the centre of the carrier lens and images of objects off the axis of the carrier fall on the peripheral retina) is very different from those for foveal vision (rotating eye, when the eye rotates to fixate objects off the carrier axis through the periphery of the multisegment lens, so that their images fall on the fovea). Ignoring the intended effects of the lenslet powers, the former can be considered to have poor optics while the latter has relatively good optics.

The intention of the multisegment lenses is that wearers should normally look through the clear central areas, with the treatment for myopia being provided by the lenslets viewed in peripheral vision. When a lens is considered by itself with an image surface being a sphere centred on the ocular pupil, the peripheral corrections are higher than for the rotating eye situation, for example, the distance part of the −4 D lens shows a cylinder of +1.3 DC × 180. The ideal shape of lenses for peripheral vision is a few dioptres steeper than for the rotating eye situation.^5^ Of course, it is not that meaningful to consider the lens alone, as unlike the rotating eye situation with foveal vision, the optics of the eye are critical. Here, the effects of the lens and eye separately are similar to their combination, at least for the presented case (Figure 5). Adding the eye gives very high levels of cylinder in the periphery, and for distance vision, it is interesting that it is in the opposite direction to the lens alone. At 32.5°, corresponding to the third hexagon, correction is −4.2 DS/−3.6 DC × 180. The additional cylinder produced through the lenslets is considerably less than that produced through the distance part of the lens. In addition to the high cylinder across the field, there is considerable variation of cylinder across the pupil (Figure 6).

For foveal vision with the rotating eye, the distance (carrier) parts of the lenses are well corrected; that is, they follow ‘best form’ principles^6^ with low levels of sagittal and tangential corrections. Adding the eye model to the lens produces only a small change in the optics, except for the addition of foveal higher order aberrations of the eye. The segments introduce some cylinder, but this is <1 D for a wide range of angles (Figure 6). Power variation across a large pupil is small, separately for distance and lenslet parts (Figure 7).

Using a 4 D accommodating version of the model eye, designed to have similar peripheral corrections as the unaccommodated eyes,^4^ comparable results were obtained in peripheral vision with an object at the near point as for a distant object with the unaccommodated eye (Figure 9).

In this study, power corrections were determined in two ways, one relative to the second principal plane of the system, and the other as conventional corrections as if a correcting lens was placed in front of the multisegment lens. As can be seen in Figure 4, the approach makes some difference to the results, with those referenced to the front of the lens being more positive (or less negative) than those referenced to the principal plane.

There are other multisegment lenses, such as the Essilor Stellest (essilor.com) and the Zeiss MyoCare (zeiss.com). The former uses rings of lenslets rather than a hexagonal arrangement, and the latter uses cylindrical rings. Analysis of these lenses will be presented in a further paper.

## CONCLUSION

Optical quality of multisegment lenses, used for the treatment of myopia, is affected considerably by oblique incidence at the lens and eye. Peripheral vision, with the eye looking through the centre of the lens, produces high levels of cylinder, both through the distance and segment parts of the lenses. Much smaller effects occur in foveal vision of the rotating eye. The present findings support the results reported earlier for the form of point images under similar conditions and emphasise the potential importance of these oblique effects in relation to possible mechanisms underlying the use of multisegment lenses for myopia control.^2^

## AUTHOR CONTRIBUTIONS

**David A. Atchison:** Conceptualization (equal); formal analysis (equal); investigation (equal); methodology (equal); resources (equal); writing – original draft (equal). **W. Neil Charman:** Conceptualization (equal); investigation (equal); writing – original draft (equal). **Matt Jaskulski:** Methodology (equal); writing – review and editing (equal).

## FUNDING INFORMATION

None.

## CONFLICT OF INTEREST STATEMENT

Matt Jaskulski: Co‐founder and CEO of VisionApp Solutions S.L., which is a start‐up company developing mobile software for vision. The remaining authors declare that they have no conflicts of interest.
